# Keeping an Eye on Bisphosphonate Therapy in Myeloma: A Case Report of Ocular Inflammation Postzoledronic Acid Infusion

**DOI:** 10.1155/2021/6647277

**Published:** 2021-02-13

**Authors:** Rehman Faryal, Amjad Hayat

**Affiliations:** Haematology Department, University Hospital Galway, Galway, Ireland

## Abstract

Bisphosphonates have evolved over the past decades from oral to more potent intravenous preparations. Along with significant paradigm shift in the management of myeloma over the past years, stronger nitrogen-containing bisphosphonates, due to their antiresorptive action on the bones, have found their way as a key and integral part in the management of bone disease in myeloma. Multiple randomized controlled trials have established efficacy of bisphosphonates in reducing skeletal-related events in myeloma. Some well-documented adverse events include acute-phase reactions, esophageal irritation, and osteonecrosis of the jaw. Across all clinical indications, the incidence of inflammatory eye reactions after bisphosphonate infusion ranges from 0.046% to 1%. However, data from myeloma patients are extrapolated from few reported cases in literature with varying management strategies including discontinuation, switching to different forms, and rechallenging with steroid cover. Inflammatory eye reactions can vary from self-limiting conjunctivitis and episcleritis to serious uveitis and vision-threatening orbital inflammation. We present a similar case of a patient with IgG kappa myeloma who developed flu-like symptoms followed by severe orbital inflammation within 48–72 hours after receiving zoledronic acid infusion. The patient was successfully managed with intravenous methyl prednisolone followed by oral tapering dose of steroids and discontinuation of further bisphosphonate therapy. A complete recovery was noted in a week's time.

## 1. Introduction

Multiple myeloma is a disorder of clonal plasma cells which are derived from postgerminal B cells. It accounts for around 1% of all cancers and 13% of hematological malignancies. Median age of diagnosis is around 70 years with 37% of myeloma patients younger than 65 years [1]. Alongside anemia, hypercalcemia, and renal impairment, lytic bone lesions are an important feature of myeloma, which can complicate the course of disease with severe pain and skeletal-related events (SREs) including pathological fractures and cord compression. Approximately 80% of multiple myeloma patients experience a pathological fracture over the course of their disease [2]. Introduction of immunomodulatory drugs, proteasome inhibitors, and monoclonal antibodies into the treatment algorithms have translated into better survival outcomes. Over the past decades, bisphosphonates have found central role in osteoporosis, malignancies, and Paget's disease, and now they are an integral part of the management of bone disease in myeloma. Bisphosphonates have evolved from oral therapies to more potent, dose-convenient, intravenous therapies. Generally, well tolerated, they are known to cause side effects ranging from mild acute-phase reactions, esophageal irritation, hypocalcemia, and atrial fibrillation to rare serious adverse events such as osteonecrosis of the jaw. Relatively rare adverse effects of bisphosphonate therapy are inflammatory eye reactions (IERs) ranging from conjunctivitis and episcleritis to vision-threatening uveitis, scleritis, and severe orbital inflammation. Due to their increasing indications, usage, and previously underreporting, more cases of ophthalmological adverse events have come to light in recent years.

## 2. Case Presentation

**Figure 1:**
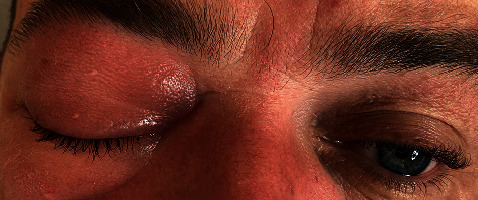
Right eyelid and periorbital swelling and redness.

A 45-year-old man presented to the eye casualty with right-sided periorbital swelling, pain, epiphora, and chemosis three days after receiving zoledronic acid in hematology day ward. He had a history of recently diagnosis of IgG kappa myeloma, R-ISS stage I, with multiple lytic lesions on the ribs, and a bone-related plasmacytoma on the left-sided ninth rib. He was an ex-smoker with a smoking history of 15 pack years. He had no other significantly past medical history and was not on any regular medications prior to his diagnosis. He was started on bortezomib/lenalidomide/dexamethasone (VRD) therapy. At the start of the second cycle, he received zoledronic acid 4 mg intravenously. He started noticing flu-like symptoms 24 hours after infusion, along with mild joint pains and minimal right eyelid swelling. Over the next 48 hours, he reported worsening right eye swelling, pain, redness, and difficulty in opening his eye (Figure 1).

**Figure 2:**
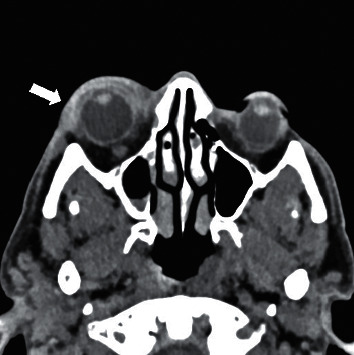
Mild right eye proptosis and soft tissue thickening overlying the right orbit (arrow). Edema of the medial and superior rectus muscles. Postseptal involvement with inflammatory fat stranding in the intraconal fat posterior to the globe.

On examination, his visual acuity was 6/9 bilaterally. A computed tomography (CT) scan of both orbits was requested to assess any possible retrobulbar inflammation. CT findings were suggestive of right-sided orbital cellulitis (Figure 2). On suspicion of a possible infective episode, he was empirically started on IV antibiotics. His symptoms continued to worsen over the next 12 hours, and it was decided to start on IV methyl prednisolone 500 mg once daily. By day three of IV steroids, there was a significant resolution of swelling and pain. He was discharged on oral prednisolone 60 mg which was tapered down every third day. He was followed up in day ward after a week where he reported complete recovery. Based on severity of the event and ophthalmology opinion, it was agreed to discontinue any further bisphosphonate therapy.

## 3. Types of Bisphosphonates and Mechanism of Action

Bisphosphonates are structural analogues of naturally occurring inorganic pyrophosphates, which bind to the hydroxyapatite binding site on the exposed areas of the bone, undergoing active resorption. During the resorptive process, bisphosphonates are absorbed by the osteoclasts, eventually leading to apoptosis.

Chemically, they are classified into two categories: nonnitrogen-containing bisphosphonates that include early first-generation bisphosphonates such as clodronate and etidronate. They are structurally very similar to naturally occurring inorganic pyrophosphates. The second category consists of nitrogen-containing bisphosphonates which include second- and third-generation compounds such as alendronate, risedronate, ibandronate, pamidronate, and zoledronic acid. The addition of nitrogen/amine group to the bisphosphonate upgrades its antiresorptive potency by 10–10,000 compared to nonnitrogen-containing bisphosphonates [3, 4]. It is important to know that, along with the antiresorptive effect, pyrophosphates (including naturally occurring inorganic pyrophosphates and bisphosphonates) also inhibit calcification and mineralization of the bone. However, the potential to inhibit mineralization differs among the bisphosphonates, for example, risedronate inhibits bone resorption and mineralization at the same concentration (therapeutic index 1 : 1). In contrast, for nitrogen-containing bisphosphonates, the potential to inhibit mineralization is 1000 times less than its antiresorptive effect at the same concentration of the drug. Therefore, it renders a favorable therapeutic index to treat conditions such as myeloma-related bone disease.

Once absorbed by the osteoclasts, nonnitrogen-containing bisphosphonates are metabolized and incorporated into newly formed adenosine triphosphate (ATP) molecules. These nonhydrolysable ATPs accumulate and are unable to drive ATP-dependent cellular processes, resulting in osteoclast apoptosis. In comparison, nitrogen-containing bisphosphonates, once absorbed by the osteoclasts, inhibit the activity of farnesyl pyrophosphate synthase, a key regulatory enzyme in the mevalonic acid pathway resulting in posttranslational modifications of key proteins and eventually resulting in osteoclast apoptosis [4].

## 4. Pathophysiology of Ocular Inflammation

Bisphosphonate treatment has been known to trigger the release of cytokines, IL-1 and IL-6, and other acute-phase proteins. In one study, it is suggested that nitrogen-containing bisphosphonates stimulate gamma/delta T cells (*γ*/*δ* T cells) in peripheral blood. Furthermore, it is suggested that the intensity of the acute-phase reaction seemed to correlate with the magnitude of increase in *γ*/*δ* T cells [5].

The exact mechanism of underlying ocular inflammatory response largely remains unknown. One suggested mechanism is that the drug is secreted into the tears by the lacrimal gland triggering a transitory localized irritation leading to the release of cytokines and other acute-phase proteins in the eye or cause activation of gamma/delta T cells within the orbit [6–8]. Preclinical animal studies reported conjunctivitis and episcleral congestion in rabbits with very supratherapeutic doses of pamidronate (daily dose of 30 mg/kg for 6 months), and it was identified that, at these doses, pamidronate secrets tears [9, 10].

The acute cytokines or T-cell response could explain the immediate eye reactions that take place within a short window. However, it is difficult to explain very delayed reactions occurring after weeks or months. It is uncertain whether the type of bisphosphonates used or disease-related factors such as background inflammatory conditions, immune dysregulation, or progressive accumulation of bisphosphonates overtime within the eye play any possible role to illicit these delayed reactions [6].

## 5. Discussion

Myeloma represents a subsegment of the patients receiving bisphosphonate therapies. Over the past years, multiple randomized placebo trials have demonstrated the efficacy of clodronate, pamidronate, and zoledronic acid in reducing bone pains and skeletal-related events. One study showed zoledronic acid was found to be as effective as pamidronate in reducing pain and incidence of SREs [11]. In another study, Medical Research Council (MRC) Myeloma IX trial, it not only shows zoledronic acid to be better than clodronate in reduction of SREs but also demonstrates that addition of zoledronic acid to standard first-line myeloma treatment reduced the risk of death by 16% and prolonged median overall survival by 5.5 months compared to clodronate [12]. However, a meta-analysis from Cochrane database was not able to confirm superiority of one bisphosphonate over another, but it is important to note that zoledronic acid is the only bisphosphonate to show survival benefit in placebo-controlled trials [13, 14]. Most experts and myeloma groups recommend a 2-year duration of bisphosphonate therapy, which is extrapolated from all placebo-controlled trials, in which the maximum duration of bisphosphonate therapy was 2 years.

Overall, the incidence of IERs after bisphosphonate exposure ranges from 0.046% to 1%, with onset occurring from a few hours after exposure up to more than 3 years, with a median of 3 weeks [9]. Around 1 in 10 patients receiving bisphosphonates have flu-like symptoms such as fever, arthralgia, and myalgia following the first dose. The rate of reactions reduces to half following subsequent infusions. In one study, HORIZON trial, the rate of acute-phase reactions after the third dose was suggested to be around 2.8% [9].

Although most of the data around ocular adverse effects of bisphosphonates comes from its use in broader categories of indications such as osteoporosis, Paget's disease, and solid cancers such as breast, prostate, and lung cancers, the data of myeloma patients come from few case reports in literature with variability in the management ranging from discontinuation of bisphosphonate therapy to rechallenging with the same or different forms of bisphosphonate. Maniel et al. report a recurrence of ocular symptoms including eyelid swelling, chemosis, and diplopia on rechallenging 4 months after the first episode with the same bisphosphonate (pamidronate) [15]. Similarly, Benderson et al. reports recurrence of milder symptoms after switching from zoledronic acid to pamidronate infusion with methyl prednisolone support and continued monthly pamidronate with steroid cover thereafter with minimal symptoms [16]. Gathering data from other indications for bisphosphonate, Fraunfelder and Fraunfelder reported 17 cases of unilateral scleritis associated with intravenous pamidronate which required discontinuation of the pamidronate therapy [17].

The decisions to rechallenge should be based on the severity of the adverse event and accurate diagnosis with formal evaluation by ophthalmology. Isolated conjunctivitis or episcleritis, having good prognosis, often experiences complete resolution without specific treatment after few days. Nonspecific conjunctivitis usually decreases in intensity during subsequent exposure to a bisphosphonate [17]. In context of myeloma where benefits clearly outweigh the risks in such circumstances, retreatment with or without steroid cover is generally safe. Patients presenting with severe orbital inflammation, once septic elements are excluded, should be promptly initiated on intravenous steroids. More severe adverse events such as uveitis, scleritis, and orbital inflammation can have serious and long-lasting consequences. In case of scleritis to fully resolve, bisphosphonate must be discontinued [17]. Cases such as these should be dealt with utmost care in conjunction with ophthalmology before a decision to rechallenge is made.

## 6. Conclusion

Bisphosphonate therapy for myeloma patients continues to be an integral part of myeloma management. Physicians and hematologists should be aware of these uncommon adverse events, and based on the severity of the events and in collaboration with ophthalmology support, a well-informed decision should be made in best interest of the patient.
